# Mapping the BCPNN Learning Rule to a Memristor Model

**DOI:** 10.3389/fnins.2021.750458

**Published:** 2021-12-09

**Authors:** Deyu Wang, Jiawei Xu, Dimitrios Stathis, Lianhao Zhang, Feng Li, Anders Lansner, Ahmed Hemani, Yu Yang, Pawel Herman, Zhuo Zou

**Affiliations:** ^1^State Key Laboratory of ASIC and System, School of Information Science and Technology, Fudan University, Shanghai, China; ^2^School of Electrical Engineering and Computer Science, KTH Royal Institute of Technology, Stockholm, Sweden; ^3^Department of Electrical Engineering, Technical University of Denmark, Kongens Lyngby, Denmark; ^4^Department of Mathematics, Stockholm University, Stockholm, Sweden

**Keywords:** Bayesian Confidence Propagation Neural Network (BCPNN), learning rule, memristor, nonlinear dopant drift phenomenon, synaptic state update, spiking neural networks, analog neuromorphic hardware

## Abstract

The Bayesian Confidence Propagation Neural Network (BCPNN) has been implemented in a way that allows mapping to neural and synaptic processes in the human cortexandhas been used extensively in detailed spiking models of cortical associative memory function and recently also for machine learning applications. In conventional digital implementations of BCPNN, the von Neumann bottleneck is a major challenge with synaptic storage and access to it as the dominant cost. The memristor is a non-volatile device ideal for artificial synapses that fuses computation and storage and thus fundamentally overcomes the von Neumann bottleneck. While the implementation of other neural networks like Spiking Neural Network (SNN) and even Convolutional Neural Network (CNN) on memristor has been studied, the implementation of BCPNN has not. In this paper, the BCPNN learning rule is mapped to a memristor model and implemented with a memristor-based architecture. The implementation of the BCPNN learning rule is a mixed-signal design with the main computation and storage happening in the analog domain. In particular, the nonlinear dopant drift phenomenon of the memristor is exploited to simulate the exponential decay of the synaptic state variables in the BCPNN learning rule. The consistency between the memristor-based solution and the BCPNN learning rule is simulated and verified in Matlab, with a correlation coefficient as high as 0.99. The analog circuit is designed and implemented in the SPICE simulation environment, demonstrating a good emulation effect for the BCPNN learning rule with a correlation coefficient as high as 0.98. This work focuses on demonstrating the feasibility of mapping the BCPNN learning rule to in-circuit computation in memristor. The feasibility of the memristor-based implementation is evaluated and validated in the paper, to pave the way for a more efficient BCPNN implementation, toward a real-time brain emulation engine.

## 1. Introduction

In the last decade, Artificial Neural Networks (ANNs) have made rapid and significant progress in real-world applications, demonstrating outstanding performance in a wide range of pattern recognition problems such as speech recognition (Hinton et al., 2012), image classification (Ciregan et al., 2012), and natural language processing (Yin et al., 2017). Despite the great success and popularity of ANNs in data-driven computational paradigms, they have some limitations. First of all, most of the existing ANNs adopt supervised learning, requiring a large amount of labeled training data, which is different from the unsupervised and reward modulated learning mechanisms attributed to biological brains. Secondly, the most prominent learning algorithm used by ANNs is error back-propagation, which requires a high level of accuracy and is neither robust nor biologically plausible. Thirdly, the current mainstream ANN models do not account for the functionality underlying human cognition and inspiring artificial intelligence, like e.g., associative memory, temporal association, and reward-based trial-and-error learning. Unlike classical ANNs with non-spiking units, Spiking Neural Networks (SNNs) use the same event-based communication mechanism as the human brain where neurons communicate with spikes.

The Bayesian Confidence Propagation Neural Network (BCPNN) was originally derived from principles of Bayesian inference (Lansner and Ekeberg, 1989; Lansner and Holst, 1996) and was further developed into an architecture inspired by the modularity of the mammalian cortex with hypercolumn units (HCUs) and minicolumn units (MCUs). Later implementation within the framework of SNNs allowed mapping to neural and synaptic processes in the human cortex (Tully et al., 2014). Compared with other SNN models, BCPNN provides a compact and practical solution for the implementation of large-scale neural networks due to its modular, coarse-grained, and hierarchical architecture. Importantly, both reduced non-spiking and biologically detailed spiking realizations of BCPNN perform similar functions. They have been extensively used to model brain-like cognitive capabilities such as associative memory (Johansson and Lansner, 2007; Lundqvist et al., 2011), episodic memory (Chrysanthidis et al., 2021), and working memory (Fiebig and Lansner, 2017; Fiebig et al., 2020), which play a key role in human intelligence. In a broader perspective, we suggest that these advancements in simulating different aspects of human cognitive function within a system framework of brain-like BCPNN constitute a promising direction in the development of artificial general intelligence (AGI).

Furthermore, the local associative nature of the Bayesian-Hebbian BCPNN learning rule has also been leveraged in cortex-inspired neural networks built for pattern recognition problems in the machine learning domain. In particular, these recent developments were facilitated by the addition of a novel brain-like structural plasticity algorithm to build a hidden layer using the original synaptic trace variables of BCPNN in an unsupervised manner (Ravichandran et al., 2020, 2021a). Classification performance on the MNIST and Fashion-MNIST benchmark problems—98.6% and 88.9% on test sets, respectively (Ravichandran et al., 2021a)—is competitive with e.g., single-layer multi-layer perceptron (MLP) with backprop, restricted Boltzmann machine (RBM), and overcomplete autoencoder. The aforementioned unsupervised nature of the structural plasticity lends itself to the efficient use of unlabeled training examples, which has been exploited to perform semi-supervised learning with competitive results on MNIST for only 10–1,000 labeled training samples (Ravichandran et al., 2021b).

At present, BCPNN is usually implemented in high-performance computers, such as clusters (Johansson and Lansner, 2007), GPUs (Yang et al., 2020; Podobas et al., 2021), and ASICs (Stathis et al., 2020). However, these systems do not fully leverage the scalability of the modular BCPNN with its local learning since they are all based on the von Neumann architecture that separates computation and storage, which puts a high demand on computing and memory access. We observe that the ASIC implementation with its full customized architecture with the 3D-RAM, achieved three orders better efficiency compared to GPUs, but it is still many orders less efficient compared to a biological brain.

Besides overcoming the von Neumann bottleneck, the non-linearity of the memristor naturally mimics the behavior of synapses. This paper shows how these properties of memristors can be leveraged to implement the BCPNN learning rule. The long-term goal of this research is to realize a large-scale memristor-based BCPNN network that is 10–100x more efficient than ASICs. However, the research presented in this paper focuses on demonstrating the feasibility of mapping the BCPNN learning rule to an in-circuit memristor-based computation. Follow-up work to this paper will focus on addressing the non-idealities of memristors and the energy efficiency analysis.

The contributions of this work are as follows:

- The non-linearity of the memristor is exploited to emulate the synaptic traces in the BCPNN learning rule. On this basis, a memristor-based architecture for the BCPNN learning rule is proposed.
- The memristor-based design for the BCPNN learning rule is simulated and verified in Matlab. The consistency between the memristor-based solution and the reference model is validated, and the correlation coefficient is as high as 0.99.
- The analog circuit for the BCPNN learning rule is designed and implemented in the SPICE simulation environment. The SPICE simulation results demonstrate a good emulation effect for the BCPNN learning rule, and the correlation coefficient is as high as 0.98.

The rest of this paper is organized as follows: Section 2 introduces the background knowledge and details about BCPNN and the memristor. Section 3 shows the similarity between the BCPNN traces and the memristor non-linearity and demonstrates how to map the BCPNN learning rule to in-circuit memristor-based computation. Section 4 proposes the memristor-based architecture for the BCPNN learning rule and explains the corresponding analog circuit design. Section 5 presents the results of Matlab and SPICE-level simulations. Section 6 summarizes the paper and further discusses several aspects of this work. Finally, section 7 presents the prospects for future work.

## 2. Preliminaries

### 2.1. BCPNN

#### 2.1.1. BCPNN Overview

The BCPNN features a modular structure in terms of HCUs and MCUs, based on a generalization of the structure of the mammalian cortex, first described by Hubel and Wiesel (Hubel and Wiesel, 1977). In models of the mammalian cortex, an HCU module has a diameter on the order of 500 μm and comprises about 100 MCUs with 50 μm diameter. Each MCU is composed of about 100 neurons, mainly excitatory pyramidal cells and one or two local inhibitory double bouquet cells (DeFelipe et al., 2006). Activity within an HCU is regulated by lateral inhibition mediated via inhibitory basket cells. In the abstract models, it takes the form of softmax that normalizes the total HCU activity (sum of the corresponding MCU activities) to 1. The number of HCUs in the human cortex has been estimated at around two million.

**Figure 1:**
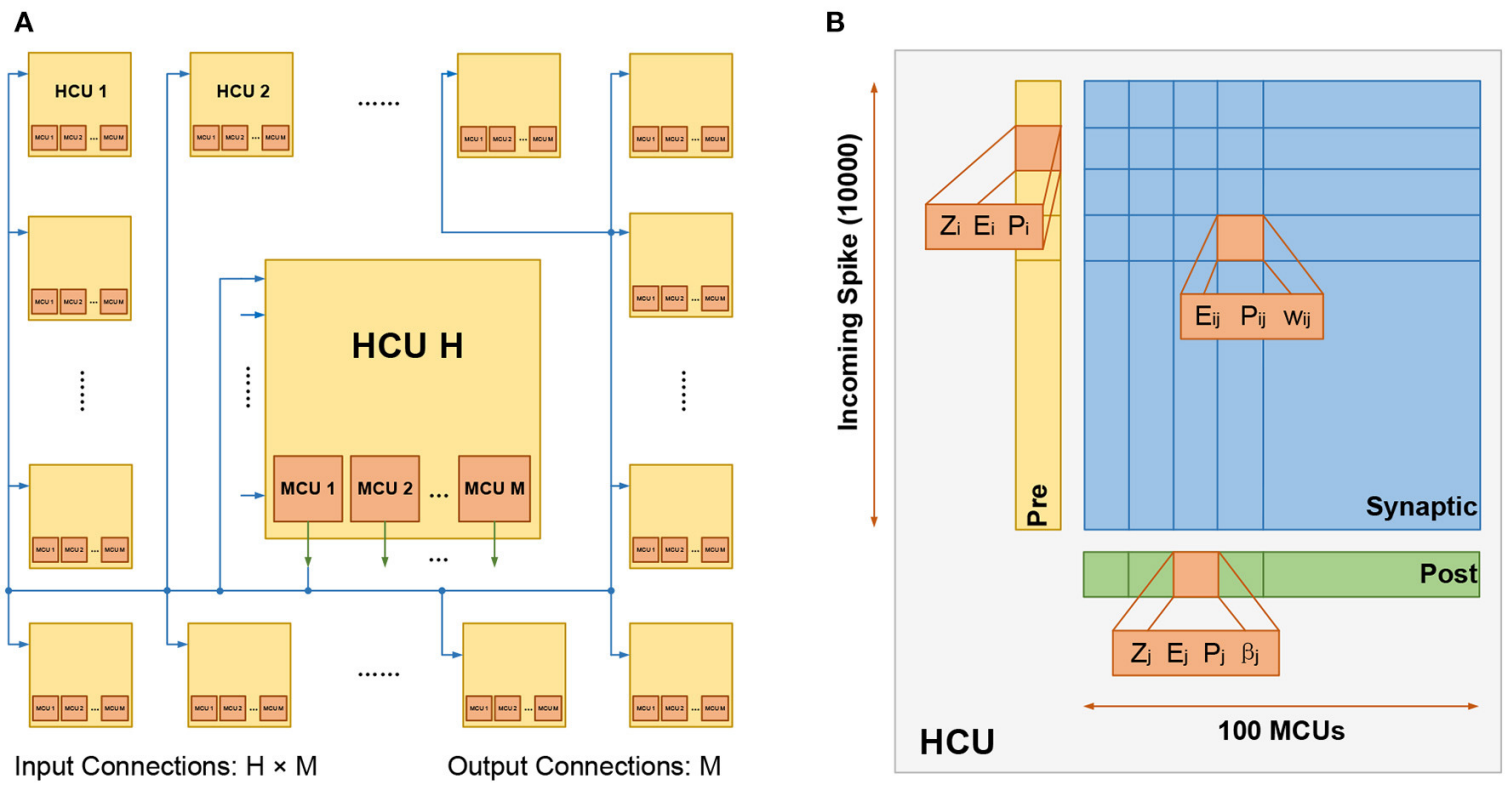
**(A)** The fully connected HCUs. The number of HCUs is *H*, each HCU contains *M* MCUs. For each HCU, the number of input connections is *H* × *M*, and the number of output connections is *M*. **(B)** The structure of HCU. The upper limit of input connections and MCUs is 10,000 and 100, respectively.

The BCPNN network can be represented with a *H* × *M* configuration, which means it is composed of *H* HCUs, and each HCU contains *M* MCUs. Generally, *H* is much larger than *M*. The number of HCUs *H* can be increased without an upper limit, while the number of MCUs *M* has an upper limit of about 100 based on biological data. Therefore, when it comes to the configuration of large networks, the number of *H* tends to be quite high. In a small network, each MCU can be fully connected to its local HCU and other HCUs, as shown in Figure 1A. Such full connectivity can not be employed in large networks due to the extreme cost of computation and storage. Instead, a cortex-inspired sparse patchy connection is adopted (Meli and Lansner, 2013), which greatly reduces the number of connections and yet maintains proper function.

Figure 1B presents the structure of the HCU, which is composed of 4 parts: 1) the presynaptic vector, used to store presynaptic traces *Z*_*i*_, *E*_*i*_ and *P*_*i*_; 2) the postsynaptic vector, used to store postsynaptic traces *Z*_*j*_, *E*_*j*_, *P*_*j*_ and the bias β_*j*_; 3) the synaptic matrix, used to store synaptic traces *E*_*ij*_, *P*_*ij*_ and the weight *w*_*ij*_. 4) a certain number of MCUs, which integrate the incoming spiking activities and fire in a soft winner-take-all manner.

At a higher level, HCUs function like independent network modules between which spikes are transmitted. The HCU size depends on the number of incoming connections and MCUs. The biologically constrained maximum number of incoming connections and MCUs is 10,000 and 100, respectively. Consequently, in a max-size HCU, a synaptic matrix with a size of 10, 000 ×100 is used, thus representing a million plastic synapses.

#### 2.1.2. BCPNN Learning Rule

**Figure 2:**
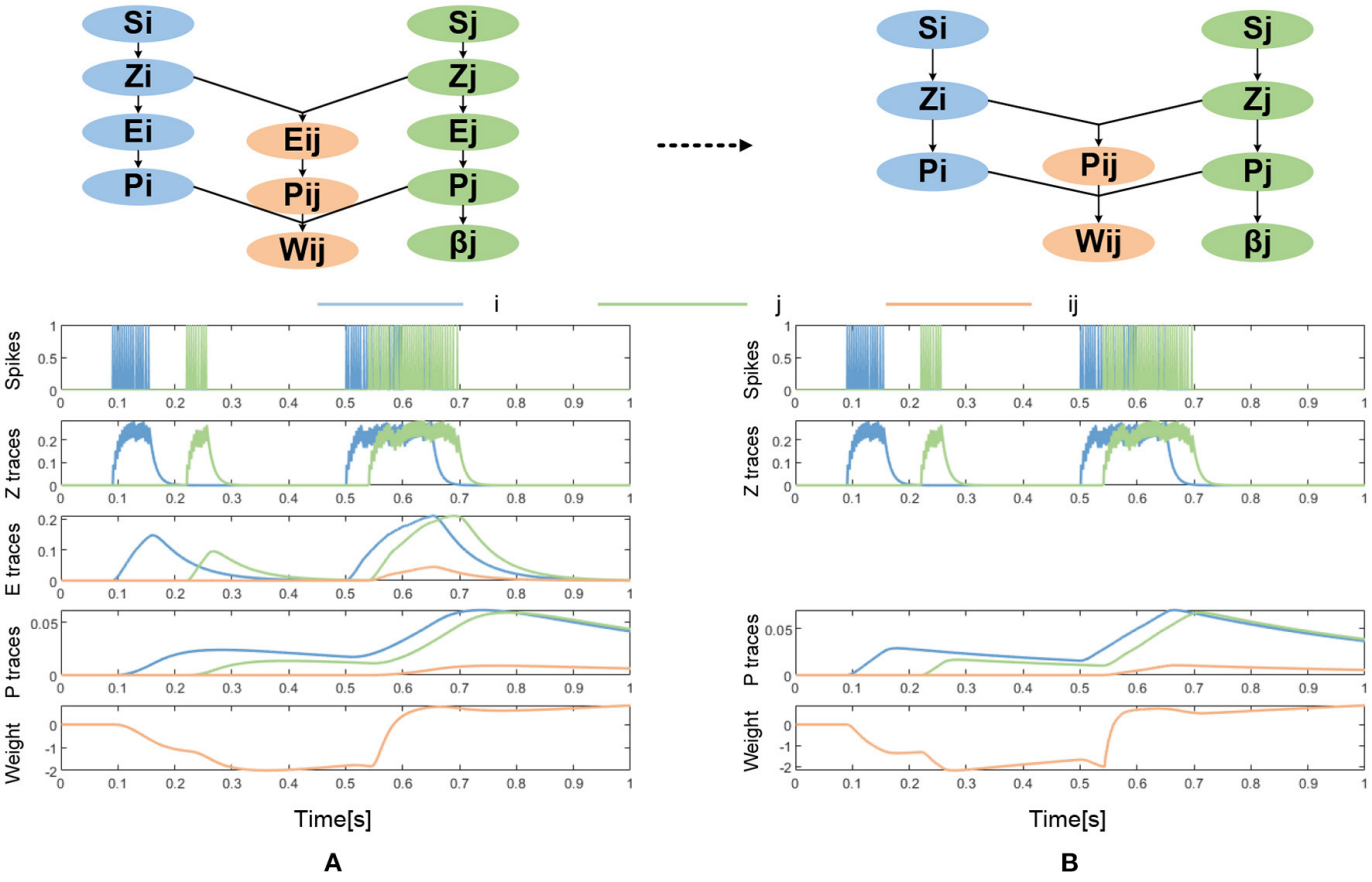
**(A)** The original BCPNN learning rule (adapted after Tully et al., 2014). **(B)** The simplified BCPNN learning rule without E trace.

The BCPNN learning rule was derived from Bayes' rule while making some independent assumptions between neural activities and by transformation to log-space to achieve a proper neural activation function (Lansner and Ekeberg, 1989; Lansner and Holst, 1996; Sandberg et al., 2002). Thus, rather than being purely phenomenological as the commonly used Spike Timing Dependent Plasticity (STDP) learning rule, it was derived from the probabilistic inference. The BCPNN learning rule is in essence another kind of Hebbian learning rule in which synaptic updates are driven by co-activation between the pre- and post-synaptic neural units. It generates positive weights if the activity between neurons is positively correlated, zero weights if they are uncorrelated, and negative weights if they are anti-correlated. Besides, it has an intrinsic bias for each neural unit which reflects the prior activation and also is observed experimentally (Tully et al., 2014). The BCPNN learning rule equations estimate the activation and co-activation of network units utilizing a cascade of three exponential running averages, as shown in Figure 2A.

First, the incoming spikes drive pre- and post-synaptic Z-traces:


(1) $$\begin{array}{r} \frac{dZ_{i}}{dt}=\frac{S_{i}-Z_{i}}{\tau_{zi}}\;\frac{dZ_{j}}{dt}=\frac{S_{j}-Z_{j}}{\tau_{zj}} \end{array}$$

Here, *i* denotes pre- and *j* denotes post-synaptic variables and S represents incoming and generated spiking activity. These Z-traces in turn drive the E-traces and P-traces following the same kind of dynamics with different time constants:


(2) $$\begin{array}{rc} \frac{dE_{i}}{dt}=\frac{Z_{i}-E_{i}}{\tau_{e}} & \frac{dE_{j}}{dt}=\frac{Z_{j}-E_{j}}{\tau_{e}}\;\frac{dE_{ij}}{dt}=\frac{Z_{i}Z_{j}-E_{ij}}{\tau_{e}} \end{array}$$

(3) $$\begin{array}{rc} \frac{dP_{i}}{dt}=\frac{\left(E_{i}-P_{i}\right)}{\tau_{p}}\kappa \;\frac{dP_{j}}{dt}=\frac{\left(E_{j}-P_{j}\right)}{\tau_{p}}\kappa & \frac{dP_{ij}}{dt}=\frac{\left(E_{ij}-P_{ij}\right)}{\tau_{p}}\kappa \end{array}$$

The learning rate κ in the dynamics of P traces modulates the learning. The E-traces form a synaptic tag important for delayed reinforcement learning. In many cases, the E-traces can be omitted and the P-traces can be updated according to equation (4) with an added parameter κ. The simplified BCPNN learning rule without E trace is shown in Figure 2B.


(4) $$\begin{array}{rc} \frac{dP_{i}}{dt}=\frac{\left(Z_{i}-P_{i}\right)}{\tau_{p}}\kappa & \frac{dP_{j}}{dt}=\frac{\left(Z_{j}-P_{j}\right)}{\tau_{p}}\kappa \;\frac{dP_{ij}}{dt}=\frac{\left(Z_{i}Z_{j}-P_{ij}\right)}{\tau_{p}}\kappa \end{array}$$

Finally, as shown in equation (5), the P-traces are used to update network unit biases, and weights with an additional parameter ε, which originates from a minimum spiking activity assumed for the pre- and postsynaptic units:


(5) $$\begin{array}{r} \beta_{j}=\log\left(P_{j}+\varepsilon \right)\;W_{ij}=\log\left(\frac{P_{ij}+\varepsilon^{2}}{\left(P_{i}+\varepsilon \right)\cdot \left(P_{j}+\varepsilon \right)}\right) \end{array}$$

#### 2.1.3. BCPNN Application and Implementation

The BCPNN model has been used for neural computation and machine learning applications as well as to model the synaptic plasticity like long-term potentiation (LTP) and long-term depression (LTD) in SNN models of cortical associative memory. In the case of neural computation, BCPNN has been used to model scalable self-organizing associative memory (Johansson and Lansner, 2007). As for the classification of the MNIST machine learning benchmarking, an accuracy of 98.6% can be achieved while maintaining a high neurobiological plausibility (Ravichandran et al., 2020, 2021a). In the latter case, the hidden layer had 200 HCUs, each with 100 MCUs. Recent cortical associative memory models have focused on synaptic working memory using BCPNN as a model for rapid cortical synaptic plasticity (Fiebig and Lansner, 2017; Fiebig et al., 2020). The positive BCPNN weights are used as excitatory connections between pyramidal cells, while the negative ones are disynaptically inhibiting pyramidal cells in distant HCUs via e.g., double bouquet cells. These SNN models are tiny compared to their biological counterparts, typically comprising up to a thousand MCUs partitioned into some 30 HCUs.

The BCPNN model has been implemented in software packages, GPU, and supercomputer clusters. It has also been implemented as custom hardware with 3D integration of DRAM for the synaptic weights (Farahini et al., 2014; Lansner et al., 2014; Stathis et al., 2020; Yang et al., 2020). The BCPNN learning rule is amenable to low-precision implementation (Vogginger et al., 2015), and the cortical memory models have proven quite robust and tolerant to external as well as to intrinsic noise and imprecision in weights and unit biases. Therefore, it is a highly scalable, modular, and hardware-friendly neuromorphic architecture with the potential for compact and low-power digital or mixed-signal design.

### 2.2. The Memristor

**Figure 3:**
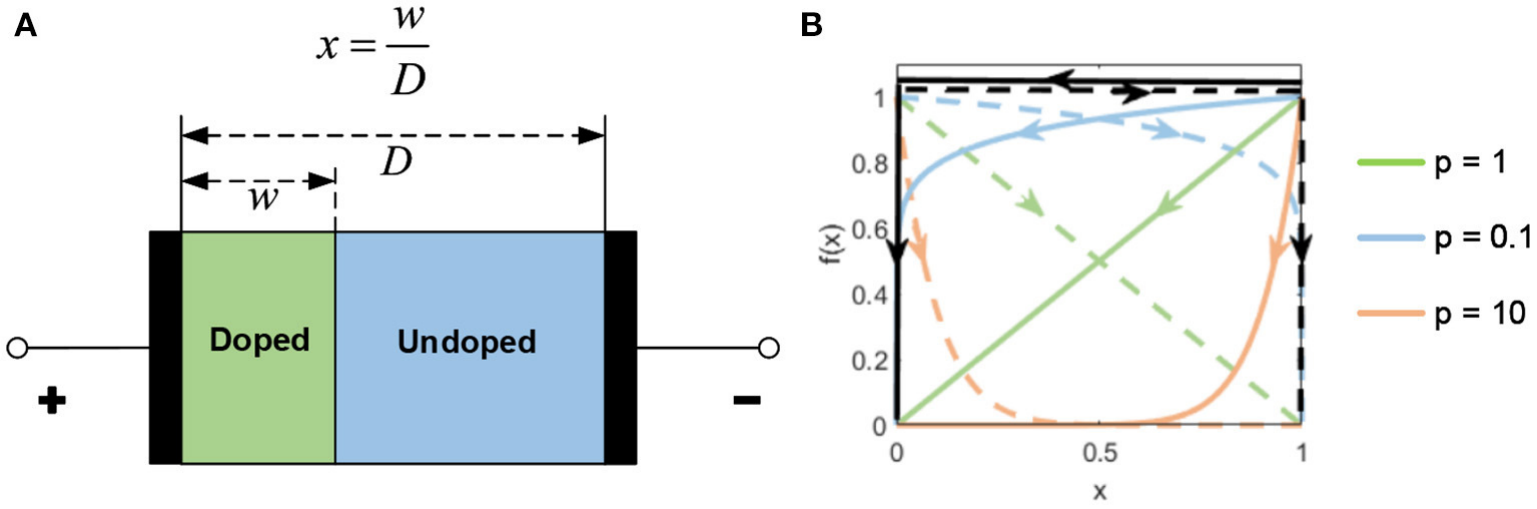
**(A)** The HP model. **(B)** The window function.

The memristor was predicted as a fourth fundamental circuit element following the resistor, capacitor, and inductor by Chua (1971). In 2008, HP Labs demonstrated and fabricated a memristor for the first time (Strukov et al., 2008). The HP Memristor was based on a nanoscale TiO_2_ thin film, with a doped region and an undoped region, as shown in Figure 3A. The total resistance of the device is determined by the variable resistances of these two regions. When an external bias voltage is applied across the device, the charged dopants will drift, moving the boundary between the two regions. The HP memristor produces rich hysteretic current-voltage behavior, which can be observed in many nanoscale electronic devices. However, in nanoscale devices, a small voltage can yield enormous electric fields, secondarily producing significant non-linearities in ionic transport, which is called the non-linear dopant drift phenomenon. This phenomenon can be represented with a window function model, as shown in Figure 3B.

The memristor has many characteristics that can be utilized in a variety of applications. To begin with, as suggested by its name, a memristive device remembers the charge that passes through it rather than storing the charge so that the memristor is nonvolatile. What is more, the memristor device can store multi-valued rather than binary values. The ability to represent multi-bit values stems from the memristor's ability to have multiple intermediate points in its transfer curve. The transfer curve, with its hysteretic behavior and ability to represent multiple values, resembles biological synapses. This is the reason for memristors attracting attention as ideal building blocks for neuromorphic structures. The ability to remember multi-valued quantities in response to voltages applied to its terminals mimics analog computation. This *in-situ* computation has also been exploited to build general-purpose computers (Zidan et al., 2018), content addressable memory (Li et al., 2020a), and to implement neural networks, both spiking and non-spiking, as discussed next.

For both non-spiking artificial neural networks and spiking neural networks, the core operation is to reinforce or weaken the synaptic weights. The algorithms used for deciding the time, magnitude, and sign of reinforcement vary from one algorithm to another. The commonality is in applying appropriate voltages for an appropriate duration to the two terminals of the memristors.

In the ANN space, several memristor-based ANNs have been studied and implemented. A single-layer perceptron (Prezioso et al., 2015) was constructed based on transistor-free metal-oxide memristor crossbars, performing the perfect classification of images. The feasibility of a three-layer fully connected neural network on MNIST and a 13-layer Convolutional Neural Network (CNN) on CIFAR-10 using the flexible memristor are studied and evaluated (Xu et al., 2018). A five-layer memristor-based CNN (Yao et al., 2020) was demonstrated to perform image recognition on MNIST, achieving an accuracy of over 96%. It is worth noting that it is challenging to take *in-situ* training on memristor-based ANNs due to non-ideal device characteristics. Prezioso's work takes *in-situ* learning with a simple learning rule called the Manhattan update rule. In Yao's work, a hybrid-training method is taken to compensate for existing device imperfections.

*In-situ* computation in memristors has also been widely studied for spiking neural networks. A supervised learning model (Nishitani et al., 2015) that enables error backpropagation for spiking neural network hardware was proposed, and the memristor was employed as an electric synapse to store the analog synaptic weight in the circuit. An all-memristor stochastic SNN architecture (Wijesinghe et al., 2018) was proposed in which the inherent stochasticity of nanoscale devices is utilized to emulate the functionality of a spiking neuron. An area-efficient memristor SNN for hardware implementation (Zhou et al., 2019) was presented based on the modified SpikeProp-like supervised learning algorithm. An STDP-based SNN (Zhao et al., 2020) was proposed to achieve the mechanism of lateral inhibition and homeostasis by memristor-based inhibitory synapses. A novel memristive synapse model based on the HP memristor was proposed, and a spiking neural network hardware fragment was constructed (Huang et al., 2021).

However, the majority of the state-of-the-art memristor-based SNNs are limited in scale and employ simple learning rules such as STDP. Compared with small-scale SNNs using STDP, the BCPNN learning rule is more complex, and its computational structure is modular and cascaded. This paper exploits the non-linearity of the memristor and elaborates how *in-situ* analog computation in the memristor has been utilized to implement the BCPNN learning rule.

## 3. A Memristor-Based BCPNN Learning Rule

### 3.1. BCPNN Model

The BCPNN learning rule has been depicted with ordinary differential equations, representing the update process of Z, E, P traces. To facilitate the hardware implementation, the ordinary differential equations (1,2,3) are further transformed to equations (6,7,8), respectively, with Euler's method, as shown below:


(6) $$\begin{array}{r} Z_{i}\left(t\right)=Z_{i}\left(t-1\right)\times \left(1-kz_{i}\right)+S_{i}\left(t-1\right)\times kz_{i}\\ Z_{j}\left(t\right)=Z_{j}\left(t-1\right)\times \left(1-kz_{j}\right)+S_{j}\left(t-1\right)\times kz_{j} \end{array}$$

(7) $$\begin{array}{r} E_{i}\left(t\right)=E_{i}\left(t-1\right)\times \left(1-ke\right)+Z_{i}\left(t-1\right)\times ke\\ E_{j}\left(t\right)=E_{j}\left(t-1\right)\times \left(1-ke\right)+Z_{j}\left(t-1\right)\times ke\\ E_{ij}\left(t\right)=E_{ij}\left(t-1\right)\times \left(1-ke\right)+Z_{i}\left(t-1\right)\times Z_{j}\left(t-1\right)\times ke \end{array}$$

(8) $$\begin{array}{r} P_{i}\left(t\right)=P_{i}\left(t-1\right)\times \left(1-kp\right)+E_{i}\left(t-1\right)\times kp\\ P_{j}\left(t\right)=P_{j}\left(t-1\right)\times \left(1-kp\right)+E_{j}\left(t-1\right)\times kp\\ P_{ij}\left(t\right)=P_{ij}\left(t-1\right)\times \left(1-kp\right)+E_{ij}\left(t-1\right)\times kp \end{array}$$

where,


(9) $$\begin{array}{r} kz_{i}=\frac{dt}{\tau_{z_{i}}}\;kz_{j}=\frac{dt}{\tau_{z_{j}}}\;ke=\frac{dt}{\tau_{e}}\;kp=\frac{dt}{\tau_{p}}\cdot \kappa \end{array}$$

The current values of Z, E, and P traces are all calculated from their previous values. The *kz*_*i*_, *kz*_*j*_, *ke* and *kp* are all constants. The simplified equation (4) can be transformed to equation (10) in the same manner, as follows:


(10) $$\begin{array}{r} P_{i}\left(t\right)=P_{i}\left(t-1\right)\times \left(1-kp\right)+Z_{i}\left(t-1\right)\times kp\\ P_{j}\left(t\right)=P_{j}\left(t-1\right)\times \left(1-kp\right)+Z_{j}\left(t-1\right)\times kp\\ P_{ij}\left(t\right)=P_{ij}\left(t-1\right)\times \left(1-kp\right)+Z_{i}\left(t-1\right)\times Z_{j}\left(t-1\right)\times kp \end{array}$$

It should be noted that we do not consider the E trace in this work to facilitate hardware implementation. Therefore, we adopt simplified equation (10), whose update process and curve of traces can be seen in Figure 2B.

### 3.2. The Memristor Model

In 2008, HP Labs proposed the linear model of a memristor (Strukov et al., 2008). Following the HP model, a variety of memristor models have been devised, such as the non-linear ion drift model (Yang et al., 2008), Simmons Tunnel Barrier Model (Pickett et al., 2009), the TEAM model (Kvatinsky et al., 2013) and the VTEAM model (Kvatinsky et al., 2015). To emulate the non-linear dopant drift phenomenon, the window function is introduced as an essential part of a memristor model, and dozens of window functions have been proposed so far. However, most window functions are facing one or more of the following problems: the boundary effect, the boundary lock, and inflexibility (Xu et al., 2021). Joglekar's window function (Joglekar and Wolf, 2009) considers the boundary effect but suffers from the boundary lock problem. Biolek's window function (Biolek et al., 2009) takes the current direction into account to solve the boundary lock issue, but its parameter setting is not flexible enough. Recently, Li's window function (Li et al., 2020b) is proposed to consider all three issues. However, Li's window function is complex, and its expression is associated with six controlling parameters, which may increase the effort required for simulation. The window function that we proposed in Xu et al. (2021) is introduced to address this problem, which is both flexible and concise.

The VTEAM model is adopted for this work because of the following advantages: 1) the VTEAM model has a good fitting effect for the nonlinear bipolar physical memristor devices that we are concerned with (Johnson et al., 2010; Chanthbouala et al., 2012; Li et al., 2018); 2) this memristor model is voltage-controlled, and the threshold voltage phenomenon has been observed in many physical devices; 3) the VTEAM model is compatible with many window functions, which demonstrates great flexibility to simulate the non-linear dopant drift phenomenon. Besides, the window function that we proposed in Xu et al. (2021) is used in this work due to its flexibility and simplicity.

The VTEAM model is shown as follows:


(11) $$\frac{dw(t)}{dt}=\left\{ \begin{array}{l} k_{\text{off}}\cdot {\left(\frac{v(t)}{v_{\text{off}}}-1\right)}^{\alpha_{\text{off}}}\cdot f(x(t)),0<v_{\text{off}}<v\\ 0,\;v_{\text{on}}<v<v_{\text{off}}\;\\ k_{\text{on}}\cdot {\left(\frac{v(t)}{v_{\text{on}}}-1\right)}^{\alpha_{\text{on}}}\cdot f(x(t)),v<v_{\text{on}}<0 \end{array}\right.$$

(12) $$\begin{array}{r} x\left(t\right)=\frac{w\left(t\right)}{W} \end{array}$$

(13) $$\begin{array}{r} R\left(t\right)=R_{\text{on}}+\left(R_{\text{off}}-R_{\text{on}}\right)\cdot x\left(t\right) \end{array}$$

(14) $$\begin{array}{r} v\left(t\right)=R\left(t\right)\cdot i\left(t\right) \end{array}$$

where *w*(*t*) is an internal state variable in [0, *W*], *W* is the maximum value of *w*(*t*), *x*(*t*) is an internal state variable in [0, 1], *f*(*x*) is the proposed window function, *v*(*t*) is the voltage across the memristor, *i*(*t*) is the current passing through the memristor, *R*(*t*) is the resistance of the memristor, and *t* is the time. The parameters *v*_on_ and *v*_off_ are threshold voltages, *R*_on_ and *R*_off_ are the corresponding resistances of the memristor when *w*(*t*) is 0 and *W*, respectively. The parameters *k*_on_, *k*_off_, α_on_ and α_off_ are constants.

The proposed window function is provided as below:


(15) $$\begin{array}{l} f(x)=j{\left[\text{sgn}(-i)\cdot (x-1)+\text{stp}(-i)\right]}^{p}\\ \text{sgn}(i)=\left\{ \begin{array}{l} 1,\;i\geq 0\\ -\text{1, }i\text{ < 0} \end{array}\right.\text{ stp}(i)=\left\{ \begin{array}{l} 1,\;i\geq 0\\ 0,\;i\text{ < 0} \end{array}\right. \end{array}$$

where *i* is the memristor current, and *j* and *p* are two tuning parameters. The memristor current *i* is positive when the internal state *x* is moving toward 1. The parameter *j* determines the magnitude, and the parameter *p* controls the decrease rate of the window function when approaching the boundaries. When *p* approaches 0, the non-linearity is weakened.

### 3.3. Similarity Between BCPNN Synaptic Traces and the Memristor Non-linearity

**Figure 4:**
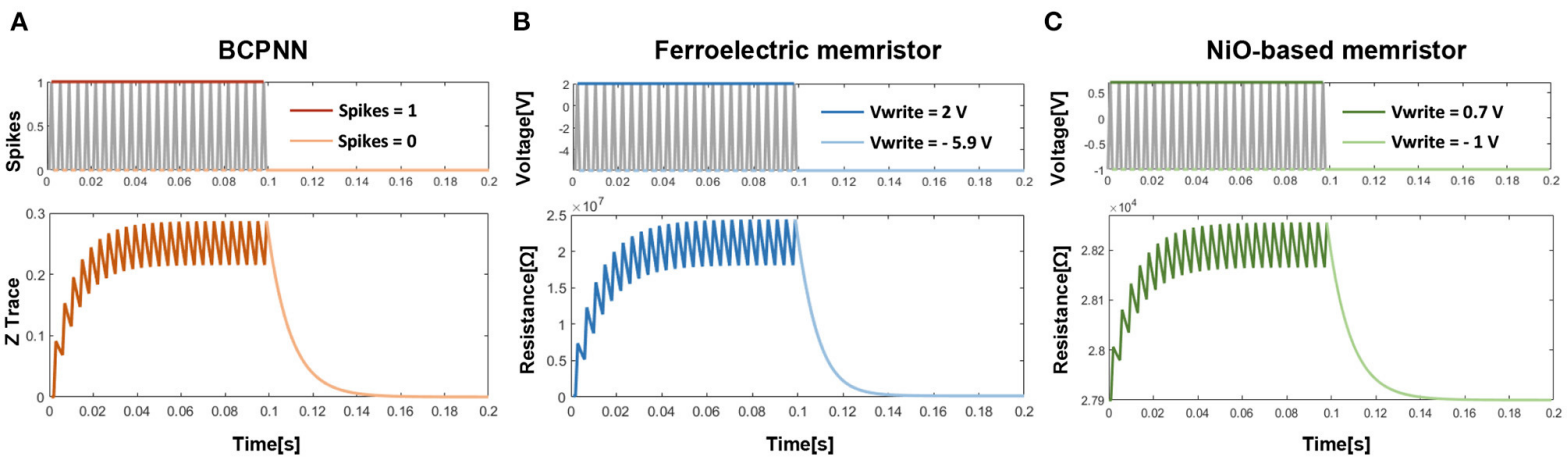
Similarity between the BCPNN trace and the memristor nonlinearity: **(A)** The curve of Z trace. **(B)** The curve of resistance of the Ferroelectrc memristor. **(C)** The curve of resistance of the NiO-based memristor.

To explore the similarity of the BCPNN traces and the memristor non-linearity, the curve of the BCPNN trace (take Z trace as an example) and the curves of the resistances of two physical memristor devices are depicted in Figure 4. As shown in Figure 4A, the Z trace of BCPNN increases when there is a spike and decreases when there is no spike. While Figures 4B,C show that the resistances of the ferroelectric memristor (Chanthbouala et al., 2012) and the NiO-based memristor (Li et al., 2018) both increase when a positive voltage is applied and decrease when a negative voltage is applied. Therefore, a similarity can be found from Figure 4 that both the BCPNN trace and the resistance of memristor change in a similar non-linear manner.

To further analyze the similarity between the BCPNN traces and the memristor non-linearity, their respective formulas are listed and compared. Take the Z trace of BCPNN as an example, when there is a spike or not, the formula of the Z trace is as follows:


(16) $$\begin{array}{r} S=1:\;Z\left(t\right)=A\cdot Z\left(t-1\right)+B\\ S=0:\;Z\left(t\right)=A\cdot Z\left(t-1\right) \end{array}$$

Correspondingly, when the voltage is positive or negative, the formula for the internal state variable of the memristor is as follows:


(17) $$\begin{array}{r} v\text{positive: }w\left(t\right)=C\cdot w\left(t-1\right)+D\\ v\text{negative: }w\left(t\right)=E\cdot w\left(t-1\right) \end{array}$$

where *A*, *B*, *C*, *D* and *E* are all constants expressed as:


(18) $$\left\{ \begin{array}{l} \text{A = 1 }-\text{ kz}\\ \text{B = kz} \end{array}\right.\left\{ \begin{array}{l} C=1-dt\cdot \frac{k_{\text{off}}}{W}\cdot {\left(\frac{v(t)}{v_{\text{off}}}-1\right)}^{\alpha_{\text{off}}}\\ \text{ D}=dt\cdot k_{\text{off}}\cdot {\left(\frac{v(t)}{v_{\text{off}}}-1\right)}^{\alpha_{\text{off}}}\\ E=1+dt.\frac{k_{\text{on}}}{W}\cdot {\left(\frac{v(t)}{v_{\text{on}}}-1\right)}^{\alpha_{\text{on}}} \end{array}\right.$$

Comparing formulas (16) and (17), a significant similarity can be observed, which also demonstrates the similarity between BCPNN traces and memristor non-linearity. Consequently, it is inspired that the non-linearity dopant drift phenomenon found in the memristor can be utilized to simulate the traces in the BCPNN learning rule.

## 4. Memristor-Based Architecture and Implementation

### 4.1. Memristor-Based Architecture

**Figure 5:**
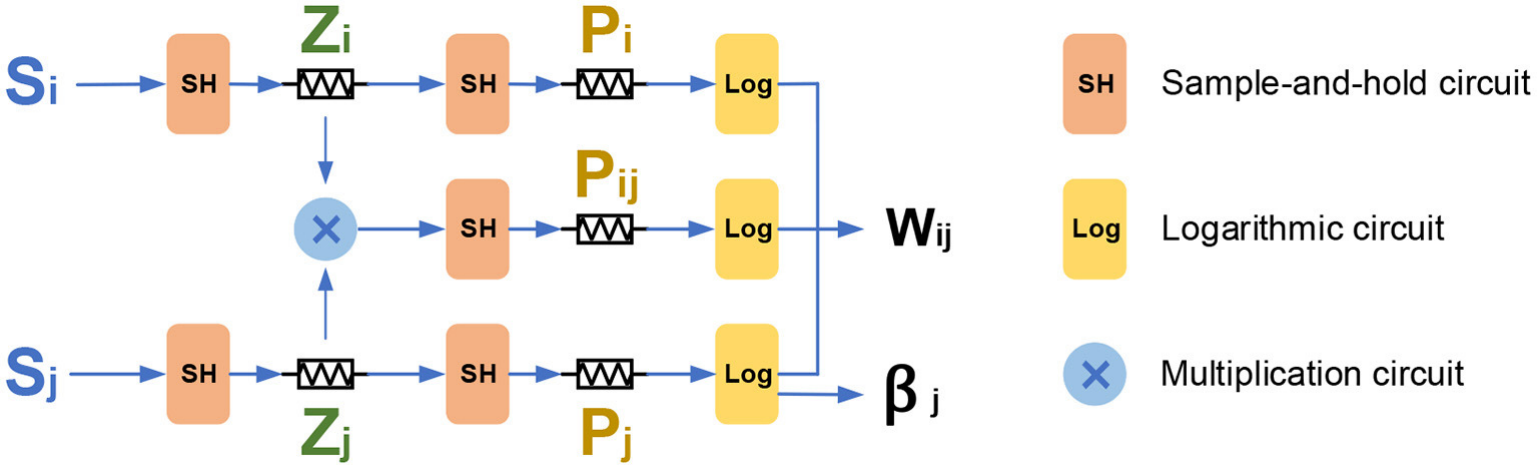
The basic memristor-based architecture for the BCPNN learning rule.

The BCPNN learning rule involves the update of synaptic traces, the bias, and the weight. Figure 5 presents the basic memristor-based architecture for the BCPNN learning rule. In the basic structure, five memristors are used to mimic the traces *Z*_*i*_, *Z*_*j*_, *P*_*i*_, *P*_*j*_, and *P*_*ij*_ respectively, and a multiplication circuit is used to calculate the product of *Z*_*i*_ and *Z*_*j*_. Besides, five sample-and-hold circuits are used to provide the converted voltage input for the memristors, and three logarithmic circuits are used to calculate the weight *w*_*ij*_ and the bias β_*j*_. The circuit design of the sample-and-hold circuit, logarithmic circuit, and the multiplication circuit will be explained in section 4.2.

As illustrated in Figure 5, the incoming presynaptic spike *S*_*i*_ is filtered into the *Z*_*i*_ trace through a sample-and-hold circuit. Then the *Z*_*i*_ trace is further filtered into the *P*_*i*_ trace with the same sample-and-hold circuit. Similarly, the postsynaptic spike *S*_*j*_ is first filtered into the *Z*_*j*_ trace, and then the *Z*_*j*_ trace is filtered into the *P*_*j*_ trace, both via a sample-and-hold circuit. Besides, the *Z*_*i*_, *Z*_*j*_ traces are multiplied with each other, and then the obtained *Z*_*i*_ × *Z*_*j*_ is filtered into the *P*_*ij*_ through a sample-and-hold circuit. Last but not least, the *P*_*ij*_, together with the *P*_*i*_ and *P*_*j*_ is used to calculate the weight *W*_*ij*_ through a logarithmic circuit. The *P*_*j*_ trace is calculated through a logarithmic circuit to obtain the value of bias β_*j*_. It should be noted that although the E trace is removed in this work, it could be added without any issue by adding another level in the cascade if the E trace is needed.

**Figure 6:**
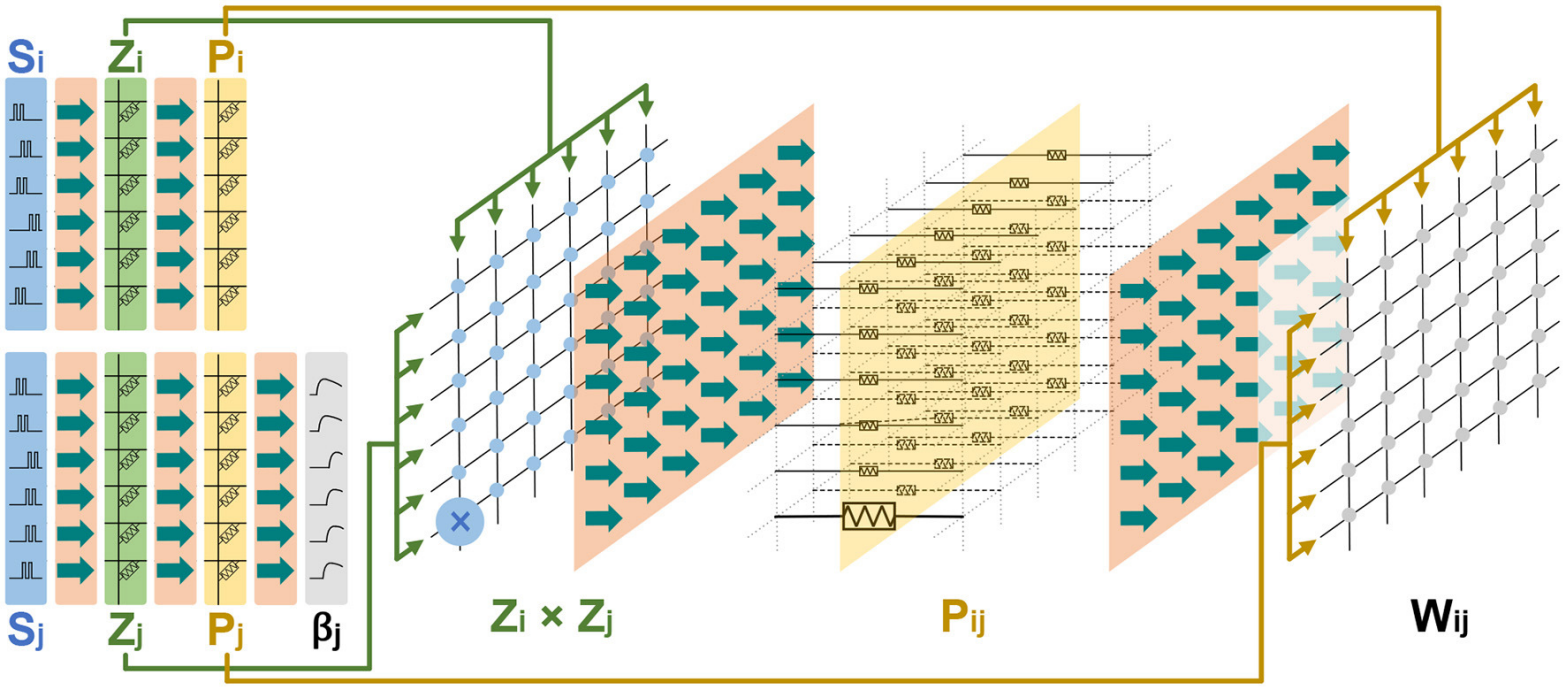
The memristor-based architecture for an HCU with a configuration of 6 × 6.

What's more, the basic memristor-based architecture described above can be reused and scaled to build a memristor-based HCU that includes more synaptic traces. As a typical case for demonstration, Figure 6 presents the memristor-based architecture for an HCU with a 6 × 6 configuration. The HCU contains a presynaptic vector of length 6, a postsynaptic vector of length 6, and a synaptic matrix of size 6 ×6.

It should be noted that the intention of Figure 6 is to illustrate the scalability of the basic architecture in Figure 5. In this work, we focus on simulating and implementing the basic memristor-based architecture for the BCPNN learning rule in Figure 5.

### 4.2. Analog Circuit Implementation

#### 4.2.1. Pre- and Post-synaptic Trace

**Figure 7:**
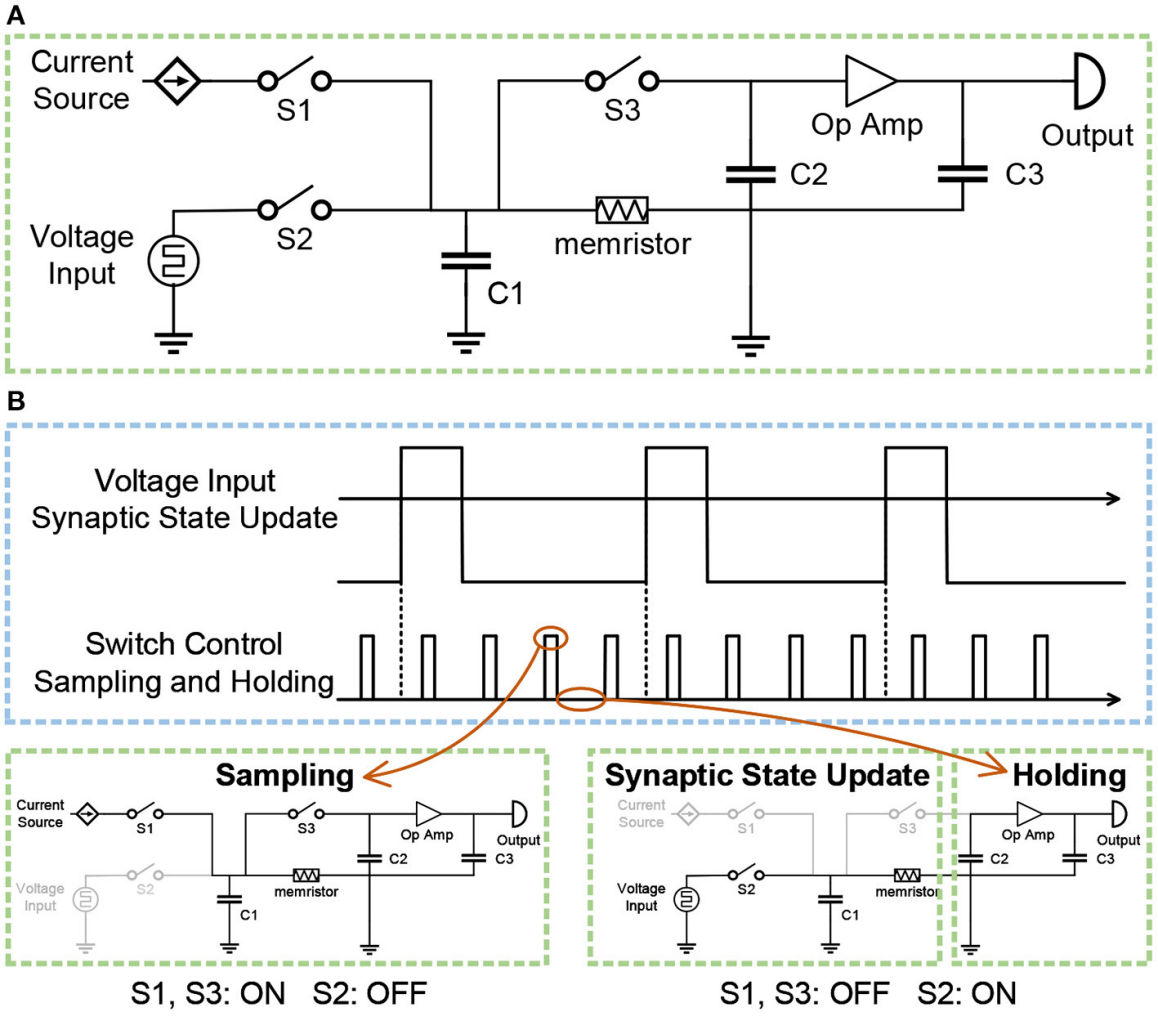
**(A)** The diagram of the sample-and-hold circuit. **(B)** The switch between the sampling state and the holding state, controlled by switches S1, S2, and S3.

The spike-based BCPNN is implemented with local synaptic state variables *Z*_*i*_, *Z*_*j*_, *P*_*i*_, *P*_*j*_ and *P*_*ij*_, which keep track of presynaptic, postsynaptic and synaptic activities. The implementation of pre- and post-synaptic trace *Z*_*i*_, *Z*_*j*_, *P*_*i*_, *P*_*j*_ can be divided into two cascaded processing stages. In the first stage, the incoming pre- and post-synaptic spike trains *S*_*i*_, *S*_*j*_ are low pass filtered into the *Z*_*i*_, *Z*_*j*_ traces, with time constants τ_*zi*_ and τ_*zj*_. In the second stage, the *Z*_*i*_, *Z*_*j*_ traces are low pass filtered into the *P*_*i*_, *P*_*j*_ traces with time constant τ_*p*_. To implement the above two cascaded processing stages, two sample-and-hold circuits are cascaded in the analog circuit implementation. Figure 7A presents the diagram of the sample-and-hold circuit. The voltage input is used to represent the incoming spike trains *S*_*i*_, *S*_*j*_. The input spike is either 0 or 1, while the voltage input is either excitatory 193.2 mV or inhibitory –149.9 mV. The input of the current source is constant, which is used to transform the resistance of the memristor into a voltage value. Switches S1, S2, S3 are used to control the switch between the sampling state and the holding state. Three capacitors C1, C2, C3, are used to store voltage. Besides, an operational amplifier is utilized to amplify the voltage value.

Figure 7B illustrates the switch between the sampling state and the holding state. When switches S1, S3 are on and switch S2 is off, the circuit is in the sampling state. The constant current of the current source passes through the memristor to obtain the voltage, which is stored in capacitor C1. Since switch S3 is on, the voltage stored in capacitor C2 is equal to the voltage stored in capacitor C1. Therefore, the resistance of the memristor is converted into the corresponding voltage value, and the voltage value is stored in the capacitor C2, thus completing a sampling process. When switch S2 is on and switches S1, S3 are off, the left part of the circuit is responsible for the update of synaptic traces, and the right part of the circuit is in the holding state. The voltage source is the input excitation of the memristor, thereby changing the resistance of the memristor. At the same time, the sampled voltage stored in the capacitor C2 is amplified by the operational amplifier, and the obtained voltage is stored in the capacitor C3 as the input voltage of the next-stage circuit. Similarly, the second stage adopts the same circuit, and the only difference is that the voltage input of the second circuit is the output of the first circuit rather than a voltage source.

#### 4.2.2. Synaptic Trace Pij

The pre- and postsynaptic traces *Z*_*i*_, *Z*_*j*_, *P*_*i*_, *P*_*j*_ can be obtained with the mentioned sample-and-hold circuit. However, to get the value of synaptic trace *P*_*ij*_, an extra multiplier is required to calculate the product of *Z*_*i*_ and *Z*_*j*_, as illustrated in formula (10).

**Figure 8:**
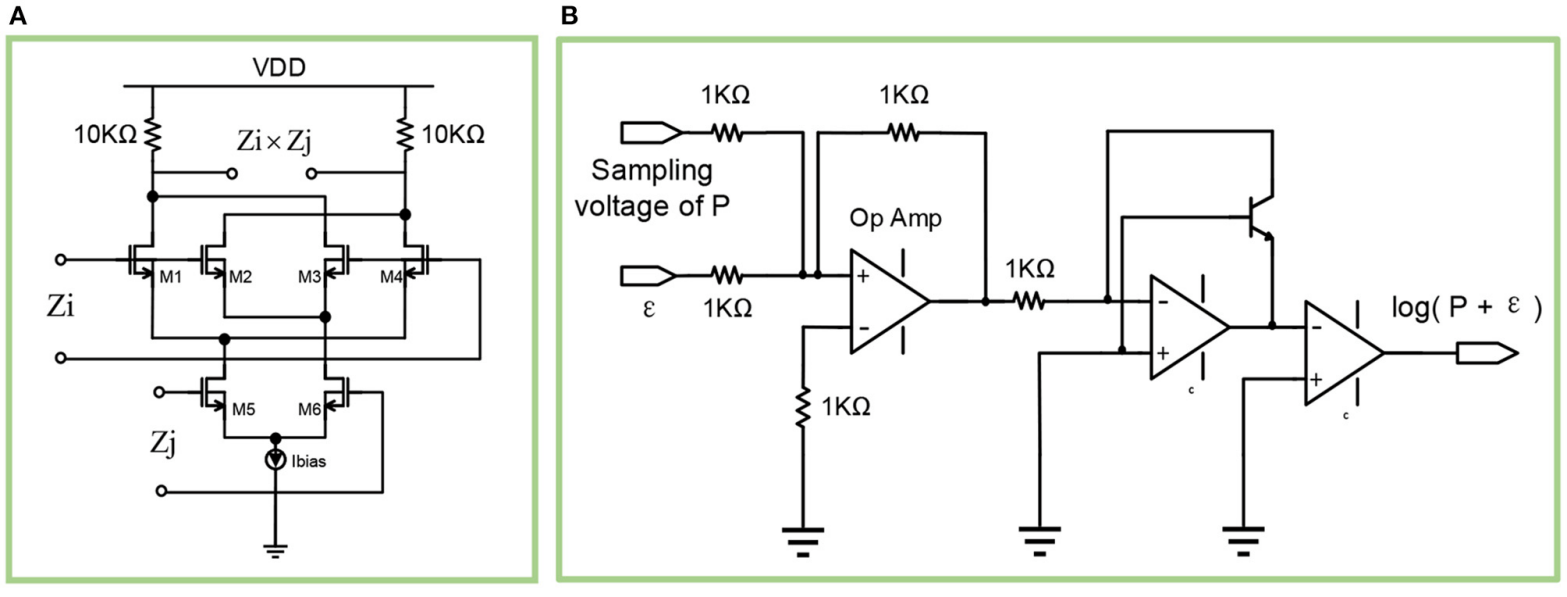
**(A)** The diagram of the multiplication circuit. **(B)** The diagram of the logarithmic circuit.

As shown in Figure 8A, the multiplication circuit is based on the classic Gilbert cell. The resistance of the two resistors are both 10kΩ. The aspect ratios *W*/*L* for *M*1, *M*2, *M*3, *M*4 are 1μm/0.18μm, while the aspect ratios *W*/*L* for *M*5, *M*6 are 2μm/0.18μm. Besides, the bias voltage Vdc for *Z*_*i*_ and *Z*_*j*_ are 1.5 v and 1.3 v respectively.

#### 4.2.3. Weight and Bias Computation

The three P traces *P*_*i*_, *P*_*j*_, *P*_*ij*_ represent the exponentially weighted moving averages of firing probability for presynaptic spikes, postsynaptic spikes, and spike co-activation respectively, which are used to compute the weight *w*_*ij*_ and the bias β_*j*_. The calculation formula (5) for *w*_*ij*_ and β_*j*_ can be further rewritten as:


(19) $$\begin{array}{l} W_{ij}=\log\left(P_{ij}+\varepsilon^{2}\right)-\log\left(P_{i}+\varepsilon \right)-\log\left(P_{j}+\varepsilon \right)\\ \beta_{j}=\log\left(P_{j}+\varepsilon \right) \end{array}$$

The key to the calculation of *w*_*ij*_ and β_*j*_ lies in the logarithmic calculation of the sum of P trace and the constant ε, as shown in formula (19). Therefore, a logarithmic calculation circuit is required. As shown in Figure 8B, the sampling voltage of P trace (*P*_*i*_, *P*_*j*_, *P*_*ij*_) is added with the constant parameter ε, then the sum is logarithmically calculated through a triode and an operational amplifier. Using such a circuit, the bias β_*j*_ can be obtained with an input pair of *P*_*i*_ and ε. Similarly, using three such circuits, whose input pairs are *P*_*ij*_ and ε^2^, *P*_*i*_ and ε, *P*_*j*_ and ε respectively, the results of the three circuits can be used to get the value of weight *w*_*ij*_.

## 5. Experimental Results

In this section, we conduct simulations to verify the feasibility of the memristor-based implementation for the BCPNN learning rule at both the algorithm level and the circuit level. From the algorithmic perspective, we conducted simulations in Matlab. From the circuit-level perspective, we conducted SPICE-level simulations. The typical values of the parameters used in the simulations are shown in Table 1, including the parameters of the BCPNN model and the memristor model.

**Table 1:** Parameters for the Simulations.

**Parameters**	**Value**	**Parameters**	**Value**
**BCPNN Model**			
*kz*_*i*_	1/11	*kz*_*j*_	1/11
*kp*	1/500	ε	0.01
**Memristor Model**			
*p*	1	*j*	1
α_off_	1	α_on_	1
*v*_off_	0.02 V	*v*_on_	–0.02 V
*R*_off_	200 kΩ	*R*_on_	2 kΩ
*k*_off_	21 nm/s	*k*_on_	–28 nm/s
*W*	1 nm	*w*_init_	0 nm
*dt*	1 ms		

### 5.1. Matlab Simulation Results

To verify the effectiveness of the memristor-based solution for the BCPNN learning rule from an algorithmic perspective, a simulation of the Z traces, P traces, the weight *w*_*ij*_, and the bias β_*j*_ is conducted using a model of the memristor device in Matlab. In the simulation, the results of the memristor-based solution are compared with those of the BCPNN reference model. The simulation lasts for 5 s with a simulation step of 1 ms, which is the simulation step in BCPNN.

**Figure 9:**
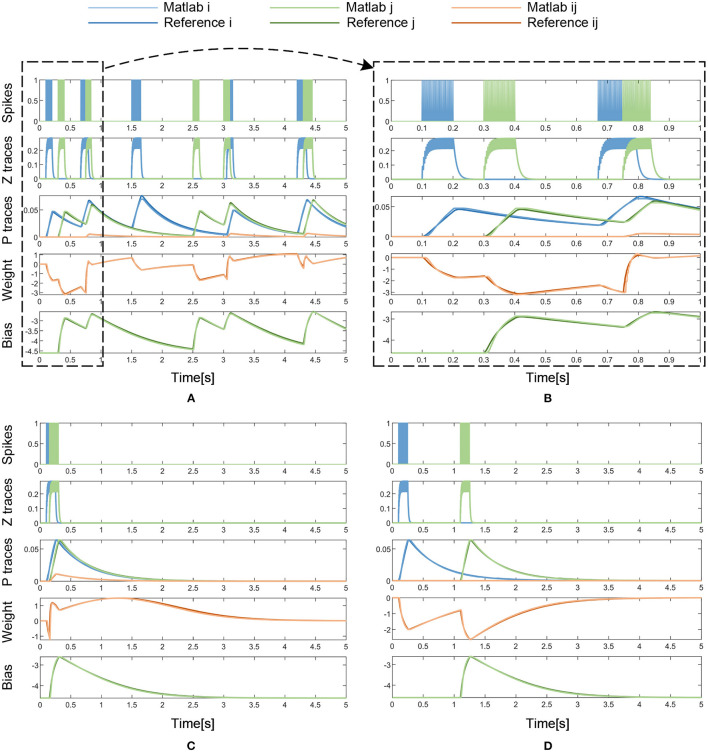
Matlab simulation results with dense or sparse incoming spikes. **(A)** Dense Spikes: 5-s simulation. **(B)** Dense Spikes: 1-s simulation. **(C)** Sparse Spikes: presynaptic spike train *S*_*i*_ overlaps with postsynaptic spike train *S*_*j*_. **(D)** Sparse Spikes: presynaptic spike train *S*_*i*_ is seperated with postsynaptic spike train *S*_*j*_.

In Figure 9 and Table 2, the simulation results are visualized and analyzed. Figure 9A presents the memristor-based 5-s Matlab simulation results with dense incoming spikes. To take a closer look at the difference between the memristor-based results and the reference model of BCPNN, the period from 0 to 1 s in Figure 9A has been enlarged, as shown in Figure 9B. With the same incoming pre- and post-synaptic spikes, the Z traces (*Z*_*i*_, *Z*_*j*_) of the memristor-based solution are the same as those of the BCPNN model. Therefore, in the Z traces part, the *Z*_*i*_, *Z*_*j*_ curves of the two models completely coincide. As for the P traces (*P*_*i*_, *P*_*j*_, *P*_*ij*_), the weight *w*_*ij*_ and the bias β_*j*_, the curves of the two models are not the same but very close. In particular, simulations with sparse spikes are carried out to observe the change of the weight in the long-lasting silent state. When the presynaptic spike train *S*_*i*_ overlaps with the postsynaptic spike train *S*_*j*_, the weight rises and finally decays to 0 in the long-lasting silent state, as shown in Figure 9C. Similarly, when the presynaptic spike train *S*_*i*_ is separated from the postsynaptic spike train *S*_*j*_, the weight drops and gradually returns to 0 in a long-lasting silent state, as shown in Figure 9D. In the analysis of the simulation results, the average error, maximum error, Root Mean Square Error (RMSE), and correlation coefficient are used as the main evaluation metrics, as shown in Table 2. Due to the nonlinearity of the memristor, the memristor-based emulation of the BCPNN learning rule is accurate with a correlation coefficient of over 0.99.

**Table 2:** Five-second simulation results with dense spikes in matlab.

**Trace**	**Mean error**	**Max error**	**RMSE**	**Correlation coefficient**
*Z*_*i*_	0.0000	0.0000	0.0000	1.0000
*Z*_*j*_	0.0000	0.0000	0.0000	1.0000
*P*_*i*_	0.0015	0.0064	0.0019	0.9961
*P*_*j*_	0.0013	0.0045	0.0015	0.9973
*P*_*ij*_	0.0001	0.0008	0.0002	0.9984
*w*_*ij*_	0.0418	1.4643	0.0862	0.9972
β_*j*_	0.0408	0.2795	0.0489	0.9979

### 5.2. SPICE Simulation Results

To further validate the feasibility of the memristor-based design from a circuit-level perspective, a SPICE-level simulation is conducted for the analog circuit implementation. In the SPICE simulation, the cascade circuit in Figure 5 is implemented, where 5 sample-and-hold circuit modules, 3 logarithmic circuit modules, and 1 multiplication circuit module described in section 4.2 are used. The parameters for the BCPNN model and the memristor model used in the SPICE simulation are the same as those used in the Matlab simulation, as shown in Table 1. It is worth noting that the timestep in the Matlab simulation is 1 ms, while the timestep in the SPICE simulation is 100 ns because of the limitation of the timestep for transistors in the simulation environment.

**Figure 10:**
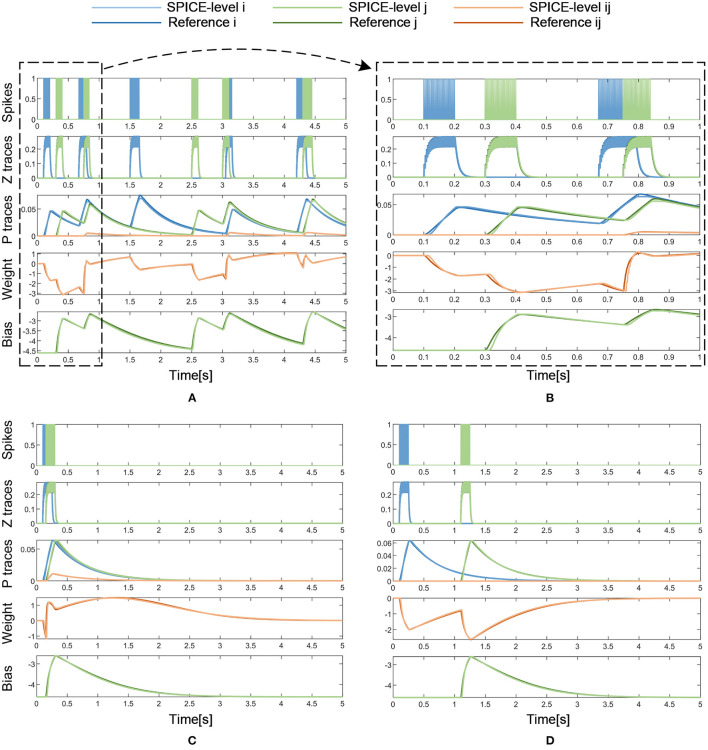
Analog circuit implementation results with dense or sparse incoming spikes. **(A)** Dense Spikes: 5-s simulation. **(B)** Dense Spikes: 1-s simulation. **(C)** Sparse Spikes: presynaptic spike train *S*_*i*_ overlaps with postsynaptic spike train *S*_*j*_. **(D)** Sparse Spikes: presynaptic spike train *S*_*i*_ is seperated with postsynaptic spike train *S*_*j*_.

With the same input of pre- and post-synaptic spikes, the results of the SPICE-level simulation are compared with those of the reference model and the error is analyzed. Figure 10A presents the SPICE simulation results with the same dense incoming spikes as in the Matlab simulation. Similarly, the period from 0 to 1 s of the simulation results is magnified to show more details of the curves, as shown in Figure 10B. Besides, Figures 10C,D also demonstrates that the weight increases with a pair of correlated *S*_*i*_ and *S*_*j*_ and decreases with a pair of uncorrelated *S*_*i*_ and *S*_*j*_. In a long-lasting silent state, the weight returns to 0 eventually. As shown in Table 3, the SPICE simulation results of memristor-based solution demonstrate a fairly high degree of fit with the reference model of BCPNN, and a correlation coefficient of over 0.98 is achieved. All in all, it is validated that the memristor-based solution for BCPNN can achieve a high degree of fit with the reference BCPNN model in the analog circuit implementation.

**Table 3:** Analog circuit implementation results of 5-s simulation with dense spikes.

**Trace**	**Mean error**	**Max error**	**RMSE**	**Correlation coefficient**
*Z*_*i*_	0.0046	0.0909	0.0147	0.9830
*Z*_*j*_	0.0041	0.0909	0.0138	0.9830
*P*_*i*_	0.0014	0.0067	0.0018	0.9965
*P*_*j*_	0.0012	0.0056	0.0015	0.9974
*P*_*ij*_	0.0001	0.0011	0.0002	0.9981
*w*_*ij*_	0.0432	1.5765	0.1003	0.9957
β_*j*_	0.0483	0.3733	0.0631	0.9971

## 6. Discussion

In this paper, the BCPNN learning rule is mapped to a memristor model and implemented with a memristor-based architecture. The similarity between the nonlinearity of the memristor and the trace update rule of BCPNN is explored and analyzed. The strong correlation between the simulated memristor-based BCPNN traces and the reference BCPNN traces has been validated in the Matlab simulation with a correlation coefficient over 0.99. Moreover, the analog circuit design of the memristor-based architecture is implemented, and the SPICE-level implementation for the BCPNN learning rule can achieve a decent emulation effect with a correlation coefficient of over 0.98.

### 6.1. Cumulative Error Analysis

The cumulative error of the memristor-based implementation can be analyzed from three aspects: the BCPNN algorithm, the memristor-based solution for BCPNN, and the analog circuit implementation. Firstly, as described before, BCPNN employs a correlation-based learning rule, which is robust and tolerant to the intrinsic noise and imprecision. BCPNN has proven to be able to function using lower precision (Vogginger et al., 2015). Secondly, as shown in Table 2, the memristor-based solution for the BCPNN learning rule presents a good simulation effect with the reference model. Moreover, it can be seen from Figure 9 that the simulation effect does not deteriorate with the increasing simulation time, which means that there is no significant increase of cumulative error. Thirdly, the same is true for the analog circuit implementation, as can be seen in Figure 10 and Table 3. Therefore, the cumulative error will not affect the stability of the memristor-based implementation for the BCPNN learning rule.

### 6.2. Setting of the Parameter ε

**Figure 11:**
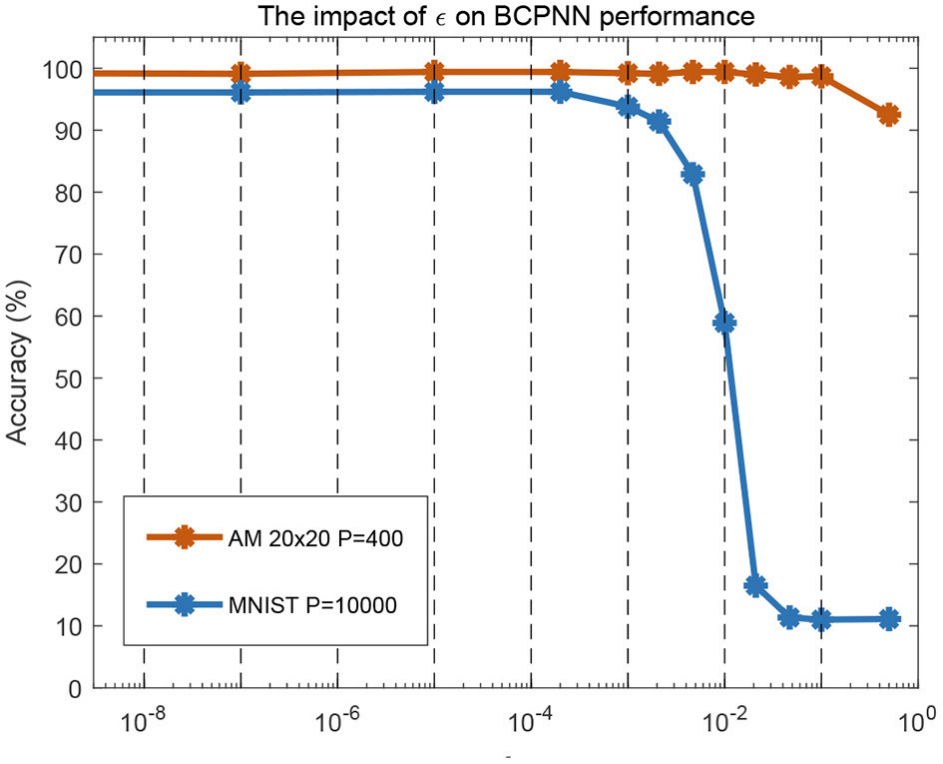
Software simulation of the impact of ε on BCPNN performance in associative memory (AM) storage capacity and handwritten digit recognition (MNIST). For AM, a network configuration of *H* × *M* = 20 × 20 was tested for storage of 400 patterns. For MNIST, all 10,000 test patterns were used.

In the BCPNN model, the setting of the parameter ε has an impact on the performance of BCPNN-based tasks, as shown in Figure 11. With ε less than 0.001, good performance was achieved in an associative memory task and a standard machine learning classification benchmark (MNIST, LeCun et al., 1998). With ε equal to 0.01, the associative memory task still maintained good performance, but the performance in the MNIST task dropped a lot. For the experiments in section 5, the parameter ε was set to be 0.01, due to the limitation of the resolution of the logarithmic circuit. Later work will seek a higher-precision analog logarithmic circuit design or adopt digital methods to implement the logarithmic calculation of weight. In this way, the value of ε can be set to be less than 0.001, which can likely meet the requirement of most BCPNN-based tasks.

It should be noted that the intention of Figure 11 is to analyze the impact of the value of ε (a parameter in the BCPNN learning rule) on the BCPNN performance from the perspective of software simulation. For the details about the working mechanism of the whole BCPNN network and how it implements associative memory tasks and practical recognition functions like MNIST classification, these works can be referred to (Johansson and Lansner, 2007; Meli and Lansner, 2013; Ravichandran et al., 2020, 2021a). The realization of the whole memristor-based BCPNN network and the algorithmic benchmarking is outside the scope of this paper and is what we plan to do in follow-up work.

### 6.3. Consideration of Device Variation

In this paper, we focus on mapping the BCPNN learning rule to a memristor model and validating the feasibility of the memristor-based implementation at the algorithm and circuit level. However, in reality, memristor-based structures suffer from device variations due to process variation and age degradation. These two factors lead to two different types of variations in the memristors devices (Park et al., 2013; Le et al., 2018). The first is spatial variations, where different devices in the crossbar react differently to the applied voltage, i.e., identical voltage pulse can drive different devices to different resistances. The second is temporal variations, where the behavior of the same device will change over time. Neural networks can adapt to such variations by taking them into account during the training of the network. This method has been used in deep neural networks (Long et al., 2019) and spiking neural networks (Querlioz et al., 2013). The authors in Querlioz et al. (2013) identify non-supervised learning as one of the fundamental benefits of the STDP learning rule that helps when dealing with device variations. Previous work indicates that the BCPNN learning rule is amenable to low-precision implementation (Vogginger et al., 2015), and the cortical memory models have proven quite robust and tolerant to external as well as to intrinsic noise and imprecision in weights and unit biases. In the follow-up work, we will focus on the non-idealities of memristors and study to what extent BCPNN's robustness can absorb the non-idealities and what other measures could be needed to cope with the non-idealities.

## 7. Future Work

As a follow-up to this paper, we plan to rigorously address the issue of nonidealities in memristors. Specifically, its variance in both space and time. We plan to quantify the extent to which BCPNN's robustness can cope with the variances and if that is not sufficient, we will study how the behavior diverges and use these experiments to devise techniques to counter the nonidealities.

Next to addressing nonidealities, the aspect on our priority list is to make the implementation more complete. This would involve implementing the control logic in CMOS, data converters, drive circuits, etc. It is also obvious, a large stack of memristor devices cannot be driven by single drivers. For this reason, we plan to experiment with and find out fragments of memristor fabrics that can be stacked with scalable drive circuits. Besides the above, we might also need to implement compensation logic to deal with nonidealities in the spirit of pre-distortion.

Having a good grip on nonidealities and more complete implementation, we will then be in a position to have a fair comparison of performance and energy efficiency between a memristor-based implementation of BCPNN and pure digital implementations that we have been experimenting with (Stathis et al., 2020; Yang et al., 2020). Besides providing realistic comparison, such an experiment will also provide us with inputs to create a more optimized implementation.

Designing memristor-based systems, at present, is a circuit-level effort. This is cumbersome and not accessible to everyone. We plan to develop, a Lego-inspired design flow called SiLago (Hemani et al., 2017), to enable automation of memristor-based designs from higher abstractions. Some work toward building such infrastructure has happened for CMOS-based conventional digital designs (Gonzalez et al., 2021; Hemani et al., 2021). We plan to enhance this for the memristors.

## Data Availability Statement

The raw data supporting the conclusions of this article will be made available by the authors, without undue reservation.

## Author Contributions

The initial idea proposed in the manuscript came from DS, AL, and AH. DW performed experiments and was responsible for the manuscript writing. JX proposed the methodology and guided the overall experimental design. LZ and FL contributed in the SPICE and Matlab simulations. ZZ provided supervision on DW, JX, and FL's work. AL, AH, DS, YY, PH, and ZZ helped with the refinement of this work and the revision of the manuscript. All authors contributed to the article and approved the submitted version.

## Funding

This work was supported in part by the National Natural Science Foundation of China under Grant 61876039 and 62011530132 (NSFC-STINT project), and Shanghai Municipal Science and Technology Major Project No. 2021SHZDZX0103 and No. 2018SHZDZX01, and in part by the Shanghai Platform for Neuromorphic and AI Chip under Grant 17DZ2260900. In part, this work was financed by the mobility grant from STINT Sweden Dnr: CH2019-8357.

## Conflict of Interest

The authors declare that the research was conducted in the absence of any commercial or financial relationships that could be construed as a potential conflict of interest.

## Publisher's Note

All claims expressed in this article are solely those of the authors and do not necessarily represent those of their affiliated organizations, or those of the publisher, the editors and the reviewers. Any product that may be evaluated in this article, or claim that may be made by its manufacturer, is not guaranteed or endorsed by the publisher.
